# 3,3,4,4,5,5-Hexafluoro-1,2-bis­(5-formyl-2-methylsulfanyl-3-thienyl)cyclo­pent-1-ene

**DOI:** 10.1107/S1600536808012695

**Published:** 2008-05-07

**Authors:** Qidong Tu, Congbin Fan, Gang Liu, Min Li, Seik Weng Ng

**Affiliations:** a School of Pharmacy, Jiangxi Science and Technology Normal University, Nanchang 330013, People’s Republic of China; b Jiangxi Key Laboratory of Organic Chemistry, Jiangxi Science and Technology Normal University, Nanchang 330013, People’s Republic of China; c Department of Chemistry, University of Malaya, 50603 Kuala Lumpur, Malaysia

## Abstract

In the crystal structure of the title diaryl­ethyl­ene compound, C_17_H_10_F_6_O_2_S_4_, the two 3-thienyl substituents are aligned at 44.9 (1) and 40.2 (1)° with respect to the –C—C=C—C– fragment of the central cyclo­pentenyl ring. The five-membered cyclo­pentenyl ring adopts an envelope conformation. The flap atom of this ring and the two F atoms bonded to it are disordered over two positions with occupancies 0.810 (5)/0.190 (5).

## Related literature

See Liu *et al.* (2008 ▶) for background literature on this class of photochromic diaryl­ethene compounds.
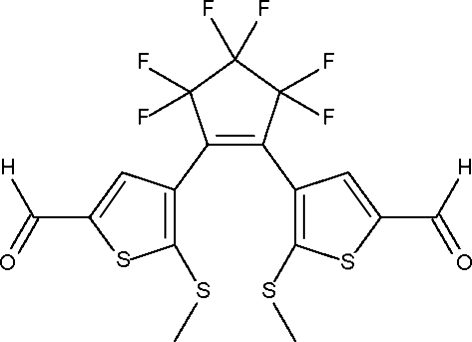


## Experimental

### 

#### Crystal data

C_17_H_10_F_6_O_2_S_4_
*M* *_r_* = 488.49
Monoclinic, 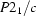

*a* = 10.7640 (8) Å
*b* = 11.8807 (9) Å
*c* = 15.486 (1) Å
β = 93.407 (1)°
*V* = 1976.9 (2) Å^3^
*Z* = 4
Mo *K*α radiation
μ = 0.55 mm^−1^
*T* = 291 (2) K
0.38 × 0.29 × 0.28 mm

#### Data collection

Bruker APEXII diffractometer
Absorption correction: multi-scan (*SADABS*; Sheldrick, 1996 ▶) *T*_min_ = 0.830, *T*_max_ = 0.858
12872 measured reflections
4488 independent reflections
3623 reflections with *I* > 2σ(*I*)
*R*_int_ = 0.017

#### Refinement

*R*[*F* ^2^ > 2σ(*F* ^2^)] = 0.048
*wR*(*F* ^2^) = 0.140
*S* = 1.03
4488 reflections
292 parameters
32 restraints
H-atom parameters constrained
Δρ_max_ = 1.28 e Å^−3^
Δρ_min_ = −0.70 e Å^−3^

### 

Data collection: *APEX2* (Bruker, 2004 ▶); cell refinement: *SAINT* (Bruker, 2004 ▶); data reduction: *SAINT*; program(s) used to solve structure: *SHELXS97* (Sheldrick, 2008 ▶); program(s) used to refine structure: *SHELXL97* (Sheldrick, 2008 ▶); molecular graphics: *X-SEED* (Barbour, 2001 ▶); software used to prepare material for publication: *publCIF* (Westrip, 2008 ▶).

## Supplementary Material

Crystal structure: contains datablocks I, global. DOI: 10.1107/S1600536808012695/xu2419sup1.cif

Structure factors: contains datablocks I. DOI: 10.1107/S1600536808012695/xu2419Isup2.hkl

Additional supplementary materials:  crystallographic information; 3D view; checkCIF report

## Figures and Tables

**Fig. 1.**
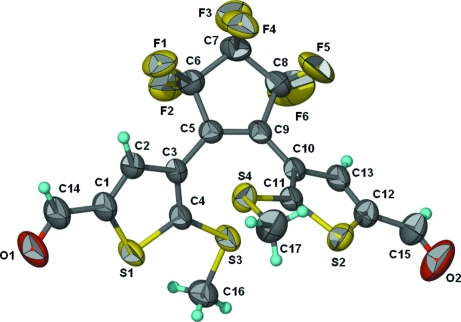
Thermal ellipsoid plot (Barbour) at the 50% probability level. Hydrogen atoms are shown as spheres of arbitrary radii; the minor disorder component is not shown.

## supplementary crystallographic information

### Comment

A previous study describes the structure of a photochromic compound, 1,2-bis[5-(4-cyanophenyl)-2-methyl-3-thienyl]-3,3,4,4,5,5-\ hexafluorocyclopent-1-ene (Liu *et al.*, 2008). The study is extended to the title compound (Scheme I, Fig. 1). The diarylethylene compound, C_17_H_10_F_6_O_2_S_4_, has its 3-thienyl substituents aligned at 41.4 (1) and 43.5 (1)° with respect to the central cyclopentenyl ring, which is almost flat.

### Experimental

5-(Methylthio)thiophene-2-carbaldehyde was brominated to 4-bromo-5-(methylthio)thiophene-2-carbaldehyde at 273 K. The carbonyl group was converted to an acetal group by the treatment of the aldehyde (3.2 g, 13.6 mmol) with glycol (4.2 g, 70 mmol) catalyzed by *p*-toluenesulfonic acid (0.07 g, 0.41 mmol) in benzene (150 ml). The resulting compound (3.82 g, 13.4 mmol) in THF (50 ml) was reacted with 2.5 *M* butyllithium (5.34 ml) at 195 K under a nitrogen atmosphere. Octafluorocyclopentene (0.91 ml, 6.68 mmol) was added, and the reaction was quenched by water. Column chromatography on silica gave 3,3,4,4,5,5-hexafluoro- 1,2-bis[5-(1,3-dioxolan-2-yl)-2-methylthio-3-thienyl]cyclopent-1-ene (1.2 g, 2.00 mmol). The compound was hydrolyzed to give the title compound in 90% yield. Yellow crystals were obtained by the vapor diffusion of chloroform and hexane (1:1). C and H elemental analysis, Calc. for C_17_H_10_F_6_O_2_S_4_ (%): C 41.80, H 2.06; found: C 41.73, H 2.07%.

### Refinement

The cyclopentenyl ring has a flat –C–C=C–C– fragment; the fifth member comprises the flap of the envelop, and is disordered. Restraints were imposed so that the ring adopts such a conformation; the 1,2-related atoms, C6/C7 and C7/C8 were restrained to 1.54±0.01 Å, and the 1,3-related C6/C8 atoms to 2.51±0.01 Å. The four C–F distances were restrained to within 0.01 Å of each other; the anisotropic temperature factors of the minor component atoms were restrained to be nearly isotropic. The disorder refined to a 81 (1):19 ratio. The final difference Fourier map had a large peak in the vicinity of the ordered fluorine atoms.

Hydrogen atoms were positioned geometrically (C–H 0.93 to 0.96 Å)] and constrained to ride on their parent atoms, with *U*_iso_(H) = 1.2 to 1.5 times the *U*_eq_ of the parent atom.

### Crystal data

**Table:**

C_17_H_10_F_6_O_2_S_4_	*F*_000_ = 984
*M**_r_* = 488.49	*D*_x_ = 1.641 Mg m^−^^3^
Monoclinic, *P*2_1_/*c*	Mo *K*α radiation λ = 0.71073 Å
Hall symbol: -P 2ybc	Cell parameters from 5293 reflections
*a* = 10.7640 (8) Å	θ = 2.6–28.2º
*b* = 11.8807 (9) Å	µ = 0.55 mm^−^^1^
*c* = 15.486 (1) Å	*T* = 291 (2) K
β = 93.407 (1)º	Block, yellow
*V* = 1976.9 (2) Å^3^	0.38 × 0.29 × 0.28 mm
*Z* = 4	

### Data collection

**Table:**

Bruker APEXII diffractometer	4488 independent reflections
Radiation source: fine-focus sealed tube	3623 reflections with *I* > 2σ(*I*)
Monochromator: graphite	*R*_int_ = 0.017
*T* = 291(2) K	θ_max_ = 27.5º
φ and ω scans	θ_min_ = 2.6º
Absorption correction: Multi-scan(SADABS; Sheldrick, 1996)	*h* = −13→13
*T*_min_ = 0.830, *T*_max_ = 0.858	*k* = −15→15
12872 measured reflections	*l* = −20→19

### Refinement

**Table:**

Refinement on *F*^2^	Secondary atom site location: difference Fourier map
Least-squares matrix: full	Hydrogen site location: inferred from neighbouring sites
*R*[*F*^2^ > 2σ(*F*^2^)] = 0.048	H-atom parameters constrained
*wR*(*F*^2^) = 0.140	*w* = 1/[σ^2^(*F*_o_^2^) + (0.0719*P*)^2^ + 1.6843*P*] where *P* = (*F*_o_^2^ + 2*F*_c_^2^)/3
*S* = 1.03	(Δ/σ)_max_ = 0.001
4488 reflections	Δρ_max_ = 1.28 e Å^−^^3^
292 parameters	Δρ_min_ = −0.70 e Å^−^^3^
32 restraints	Extinction correction: none
Primary atom site location: structure-invariant direct methods	

### Fractional atomic coordinates and isotropic or equivalent isotropic displacement parameters (Å^2^)

**Table:**

	*x*	*y*	*z*	*U*_iso_*/*U*_eq_	Occ. (<1)
S1	0.85945 (7)	0.33347 (7)	0.64145 (5)	0.0517 (2)	
S2	0.88965 (7)	−0.05788 (6)	0.39840 (5)	0.0492 (2)	
S3	0.71640 (7)	0.12007 (6)	0.59914 (4)	0.0464 (2)	
S4	0.91715 (7)	0.19624 (6)	0.38742 (5)	0.04583 (19)	
F1	0.56368 (19)	0.45268 (16)	0.37042 (15)	0.0697 (6)	
F2	0.4537 (2)	0.3805 (2)	0.46758 (14)	0.0746 (6)	
F3	0.3300 (2)	0.3052 (3)	0.3266 (3)	0.0812 (12)	0.810 (5)
F4	0.4907 (3)	0.3241 (2)	0.25152 (15)	0.0715 (10)	0.810 (5)
F5	0.4988 (3)	0.1142 (3)	0.26891 (18)	0.1259 (13)	
F6	0.4066 (2)	0.1119 (2)	0.3863 (2)	0.1093 (11)	
O1	0.9727 (3)	0.5672 (2)	0.66318 (18)	0.0827 (9)	
O2	0.8080 (3)	−0.3083 (2)	0.4030 (3)	0.0962 (10)	
C1	0.8505 (3)	0.4474 (2)	0.5720 (2)	0.0494 (7)	
C2	0.7748 (3)	0.4263 (2)	0.49994 (19)	0.0442 (6)	
H2	0.7594	0.4784	0.4557	0.053*	
C3	0.7220 (2)	0.3165 (2)	0.49939 (16)	0.0371 (5)	
C4	0.7605 (2)	0.2565 (2)	0.57284 (16)	0.0377 (5)	
C5	0.6309 (2)	0.2760 (2)	0.43218 (15)	0.0356 (5)	
C6	0.5253 (2)	0.3536 (2)	0.40199 (18)	0.0431 (6)	
C7	0.4524 (3)	0.2905 (4)	0.3281 (3)	0.0486 (11)	0.810 (5)
C8	0.4951 (3)	0.1683 (2)	0.3416 (2)	0.0504 (7)	
C9	0.6167 (2)	0.1729 (2)	0.39523 (16)	0.0358 (5)	
C10	0.6979 (2)	0.0740 (2)	0.39929 (16)	0.0364 (5)	
C11	0.8271 (2)	0.0761 (2)	0.39533 (16)	0.0376 (5)	
C12	0.7434 (3)	−0.1179 (2)	0.40298 (19)	0.0461 (6)	
C13	0.6523 (3)	−0.0383 (2)	0.40251 (17)	0.0420 (6)	
H13	0.5682	−0.0557	0.4041	0.050*	
C14	0.9139 (3)	0.5520 (3)	0.5951 (2)	0.0642 (9)	
H14	0.9091	0.6106	0.5552	0.077*	
C15	0.7257 (4)	−0.2395 (3)	0.4026 (2)	0.0620 (9)	
H15	0.6444	−0.2661	0.4020	0.074*	
C16	0.7725 (3)	0.1094 (3)	0.71059 (18)	0.0535 (7)	
H16A	0.8616	0.1157	0.7146	0.080*	
H16B	0.7485	0.0380	0.7334	0.080*	
H16C	0.7373	0.1689	0.7433	0.080*	
C17	1.0583 (3)	0.1414 (3)	0.3464 (2)	0.0593 (8)	
H17A	1.1026	0.0985	0.3908	0.089*	
H17B	1.1095	0.2026	0.3292	0.089*	
H17C	1.0380	0.0939	0.2975	0.089*	
F3'	0.3375 (10)	0.2469 (12)	0.4024 (8)	0.081 (4)	0.190 (5)
F4'	0.3867 (17)	0.3304 (12)	0.2800 (9)	0.090 (5)	0.190 (5)
C7'	0.4250 (7)	0.2804 (7)	0.3523 (7)	0.050 (6)	0.190 (5)

### Atomic displacement parameters (Å^2^)

**Table:**

	*U*^11^	*U*^22^	*U*^33^	*U*^12^	*U*^13^	*U*^23^
S1	0.0604 (5)	0.0470 (4)	0.0453 (4)	−0.0169 (3)	−0.0169 (3)	−0.0005 (3)
S2	0.0508 (4)	0.0352 (4)	0.0612 (5)	0.0014 (3)	−0.0004 (3)	0.0031 (3)
S3	0.0593 (4)	0.0397 (4)	0.0387 (3)	−0.0147 (3)	−0.0086 (3)	0.0039 (3)
S4	0.0410 (4)	0.0356 (3)	0.0608 (4)	−0.0068 (3)	0.0023 (3)	−0.0021 (3)
F1	0.0693 (12)	0.0492 (10)	0.0876 (14)	−0.0059 (9)	−0.0189 (10)	0.0269 (10)
F2	0.0707 (13)	0.0872 (16)	0.0671 (12)	0.0286 (11)	0.0134 (10)	−0.0040 (11)
F3	0.0335 (13)	0.0724 (19)	0.134 (3)	0.0019 (12)	−0.0234 (15)	−0.0018 (19)
F4	0.086 (2)	0.0789 (18)	0.0469 (13)	−0.0162 (15)	−0.0187 (12)	0.0168 (12)
F5	0.129 (2)	0.140 (2)	0.0991 (19)	0.062 (2)	−0.0753 (17)	−0.0722 (19)
F6	0.0586 (13)	0.0851 (17)	0.180 (3)	−0.0356 (12)	−0.0303 (16)	0.0536 (18)
O1	0.100 (2)	0.0659 (16)	0.0777 (17)	−0.0379 (15)	−0.0308 (15)	−0.0051 (13)
O2	0.102 (2)	0.0389 (13)	0.147 (3)	0.0066 (14)	0.004 (2)	0.0123 (16)
C1	0.0559 (17)	0.0376 (14)	0.0532 (16)	−0.0134 (12)	−0.0079 (13)	−0.0022 (12)
C2	0.0512 (16)	0.0328 (13)	0.0474 (15)	−0.0053 (11)	−0.0067 (12)	0.0013 (11)
C3	0.0411 (13)	0.0315 (12)	0.0381 (12)	−0.0041 (10)	−0.0035 (10)	−0.0036 (10)
C4	0.0403 (13)	0.0349 (12)	0.0375 (12)	−0.0070 (10)	−0.0034 (10)	−0.0034 (10)
C5	0.0357 (12)	0.0345 (12)	0.0361 (12)	−0.0035 (10)	−0.0019 (9)	0.0020 (10)
C6	0.0421 (14)	0.0399 (14)	0.0469 (15)	0.0008 (11)	−0.0008 (11)	0.0027 (11)
C7	0.0330 (18)	0.057 (2)	0.054 (3)	−0.0033 (17)	−0.007 (2)	0.0045 (19)
C8	0.0456 (16)	0.0448 (15)	0.0584 (17)	−0.0084 (12)	−0.0162 (13)	−0.0051 (13)
C9	0.0360 (12)	0.0358 (12)	0.0350 (12)	−0.0067 (10)	−0.0037 (9)	0.0002 (10)
C10	0.0432 (14)	0.0316 (12)	0.0334 (12)	−0.0063 (10)	−0.0047 (10)	−0.0013 (9)
C11	0.0428 (14)	0.0331 (12)	0.0364 (12)	−0.0040 (10)	−0.0038 (10)	−0.0006 (10)
C12	0.0565 (17)	0.0343 (13)	0.0468 (15)	−0.0083 (12)	−0.0022 (12)	0.0022 (11)
C13	0.0467 (15)	0.0362 (13)	0.0424 (14)	−0.0100 (11)	−0.0044 (11)	−0.0010 (11)
C14	0.076 (2)	0.0452 (17)	0.069 (2)	−0.0222 (16)	−0.0169 (17)	−0.0023 (15)
C15	0.080 (2)	0.0343 (15)	0.070 (2)	−0.0059 (15)	−0.0033 (17)	0.0079 (14)
C16	0.0669 (19)	0.0532 (17)	0.0394 (14)	−0.0042 (15)	−0.0055 (13)	0.0047 (13)
C17	0.0416 (16)	0.0531 (18)	0.084 (2)	−0.0022 (13)	0.0075 (15)	0.0001 (16)
F3'	0.048 (5)	0.102 (8)	0.093 (8)	−0.017 (5)	0.007 (5)	−0.019 (6)
F4'	0.083 (9)	0.094 (8)	0.086 (7)	0.014 (7)	−0.043 (7)	0.001 (6)
C7'	0.057 (10)	0.053 (8)	0.038 (8)	−0.001 (7)	−0.002 (7)	0.014 (6)

### Geometric parameters (Å, °)

**Table:**

S1—C4	1.722 (2)	C5—C6	1.516 (4)
S1—C1	1.728 (3)	C6—C7	1.543 (4)
S2—C11	1.728 (3)	C6—C7'	1.554 (8)
S2—C12	1.734 (3)	C7—C8	1.534 (5)
S3—C4	1.744 (3)	C8—C9	1.509 (4)
S3—C16	1.799 (3)	C8—C7'	1.545 (8)
S4—C11	1.734 (3)	C9—C10	1.463 (4)
S4—C17	1.803 (3)	C10—C11	1.397 (4)
F1—C6	1.349 (3)	C10—C13	1.424 (3)
F2—C6	1.349 (3)	C12—C13	1.362 (4)
F3—C7	1.329 (4)	C12—C15	1.456 (4)
F4—C7	1.339 (5)	C13—H13	0.9300
F5—C8	1.298 (4)	C14—H14	0.9300
F6—C8	1.384 (4)	C15—H15	0.9300
O1—C14	1.211 (4)	C16—H16A	0.9600
O2—C15	1.205 (4)	C16—H16B	0.9600
C1—C2	1.364 (4)	C16—H16C	0.9600
C1—C14	1.453 (4)	C17—H17A	0.9600
C2—C3	1.423 (3)	C17—H17B	0.9600
C2—H2	0.9300	C17—H17C	0.9600
C3—C4	1.385 (4)	F3'—C7'	1.317 (8)
C3—C5	1.468 (3)	F4'—C7'	1.313 (8)
C5—C9	1.356 (3)		
			
C4—S1—C1	91.26 (13)	F6—C8—C7'	90.5 (5)
C11—S2—C12	91.55 (13)	C9—C8—C7'	108.8 (3)
C4—S3—C16	102.05 (14)	C5—C9—C10	130.9 (2)
C11—S4—C17	102.34 (14)	C5—C9—C8	109.7 (2)
C2—C1—C14	127.4 (3)	C10—C9—C8	119.3 (2)
C2—C1—S1	112.0 (2)	C11—C10—C13	111.4 (2)
C14—C1—S1	120.6 (2)	C11—C10—C9	125.3 (2)
C1—C2—C3	113.1 (2)	C13—C10—C9	123.3 (2)
C1—C2—H2	123.5	C10—C11—S2	111.69 (19)
C3—C2—H2	123.5	C10—C11—S4	125.5 (2)
C4—C3—C2	111.5 (2)	S2—C11—S4	122.82 (16)
C4—C3—C5	124.7 (2)	C13—C12—C15	126.5 (3)
C2—C3—C5	123.7 (2)	C13—C12—S2	111.7 (2)
C3—C4—S1	112.20 (19)	C15—C12—S2	121.8 (3)
C3—C4—S3	126.69 (19)	C12—C13—C10	113.7 (2)
S1—C4—S3	121.10 (15)	C12—C13—H13	123.2
C9—C5—C3	130.4 (2)	C10—C13—H13	123.2
C9—C5—C6	110.8 (2)	O1—C14—C1	123.8 (3)
C3—C5—C6	118.5 (2)	O1—C14—H14	118.1
F1—C6—F2	105.5 (2)	C1—C14—H14	118.1
F1—C6—C5	113.7 (2)	O2—C15—C12	125.2 (4)
F2—C6—C5	111.3 (2)	O2—C15—H15	117.4
F1—C6—C7	107.9 (3)	C12—C15—H15	117.4
F2—C6—C7	112.7 (3)	S3—C16—H16A	109.5
C5—C6—C7	105.8 (2)	S3—C16—H16B	109.5
F1—C6—C7'	121.9 (4)	H16A—C16—H16B	109.5
F2—C6—C7'	95.4 (4)	S3—C16—H16C	109.5
C5—C6—C7'	107.4 (3)	H16A—C16—H16C	109.5
F3—C7—F4	107.7 (3)	H16B—C16—H16C	109.5
F3—C7—C8	114.6 (3)	S4—C17—H17A	109.5
F4—C7—C8	107.3 (3)	S4—C17—H17B	109.5
F3—C7—C6	114.1 (4)	H17A—C17—H17B	109.5
F4—C7—C6	110.1 (3)	S4—C17—H17C	109.5
C8—C7—C6	102.9 (2)	H17A—C17—H17C	109.5
F5—C8—F6	104.6 (3)	H17B—C17—H17C	109.5
F5—C8—C9	115.2 (3)	F4'—C7'—F3'	116.1 (12)
F6—C8—C9	110.0 (2)	F4'—C7'—C8	115.4 (10)
F5—C8—C7	112.0 (3)	F3'—C7'—C8	100.0 (8)
F6—C8—C7	108.5 (3)	F4'—C7'—C6	110.0 (9)
C9—C8—C7	106.5 (2)	F3'—C7'—C6	112.1 (8)
F5—C8—C7'	124.0 (4)	C8—C7'—C6	101.9 (4)
			
C4—S1—C1—C2	0.1 (3)	F5—C8—C9—C5	140.8 (3)
C4—S1—C1—C14	177.1 (3)	F6—C8—C9—C5	−101.3 (3)
C14—C1—C2—C3	−176.8 (3)	C7—C8—C9—C5	16.0 (4)
S1—C1—C2—C3	0.0 (4)	C7'—C8—C9—C5	−3.7 (5)
C1—C2—C3—C4	−0.1 (4)	F5—C8—C9—C10	−38.5 (4)
C1—C2—C3—C5	175.6 (3)	F6—C8—C9—C10	79.4 (3)
C2—C3—C4—S1	0.1 (3)	C7—C8—C9—C10	−163.3 (3)
C5—C3—C4—S1	−175.5 (2)	C7'—C8—C9—C10	177.0 (5)
C2—C3—C4—S3	178.9 (2)	C5—C9—C10—C11	−41.3 (4)
C5—C3—C4—S3	3.2 (4)	C8—C9—C10—C11	137.8 (3)
C1—S1—C4—C3	−0.1 (2)	C5—C9—C10—C13	142.7 (3)
C1—S1—C4—S3	−178.93 (19)	C8—C9—C10—C13	−38.2 (4)
C16—S3—C4—C3	−167.9 (3)	C13—C10—C11—S2	−1.5 (3)
C16—S3—C4—S1	10.8 (2)	C9—C10—C11—S2	−177.9 (2)
C4—C3—C5—C9	−41.9 (4)	C13—C10—C11—S4	178.00 (19)
C2—C3—C5—C9	142.9 (3)	C9—C10—C11—S4	1.6 (4)
C4—C3—C5—C6	131.4 (3)	C12—S2—C11—C10	1.0 (2)
C2—C3—C5—C6	−43.8 (4)	C12—S2—C11—S4	−178.51 (18)
C9—C5—C6—F1	−127.5 (3)	C17—S4—C11—C10	−160.0 (2)
C3—C5—C6—F1	58.0 (3)	C17—S4—C11—S2	19.5 (2)
C9—C5—C6—F2	113.5 (3)	C11—S2—C12—C13	−0.3 (2)
C3—C5—C6—F2	−61.0 (3)	C11—S2—C12—C15	176.7 (3)
C9—C5—C6—C7	−9.2 (3)	C15—C12—C13—C10	−177.4 (3)
C3—C5—C6—C7	176.3 (3)	S2—C12—C13—C10	−0.6 (3)
C9—C5—C6—C7'	10.3 (5)	C11—C10—C13—C12	1.4 (3)
C3—C5—C6—C7'	−164.2 (5)	C9—C10—C13—C12	177.8 (2)
F1—C6—C7—F3	−95.2 (4)	C2—C1—C14—O1	174.5 (4)
F2—C6—C7—F3	20.9 (5)	S1—C1—C14—O1	−2.0 (6)
C5—C6—C7—F3	142.8 (3)	C13—C12—C15—O2	180.0 (4)
C7'—C6—C7—F3	45.2 (10)	S2—C12—C15—O2	3.4 (5)
F1—C6—C7—F4	25.9 (4)	F5—C8—C7'—F4'	−12.0 (12)
F2—C6—C7—F4	142.0 (3)	F6—C8—C7'—F4'	−120.0 (10)
C5—C6—C7—F4	−96.1 (3)	C9—C8—C7'—F4'	128.6 (10)
C7'—C6—C7—F4	166.3 (11)	C7—C8—C7'—F4'	42.7 (11)
F1—C6—C7—C8	140.1 (3)	F5—C8—C7'—F3'	113.4 (7)
F2—C6—C7—C8	−103.8 (3)	F6—C8—C7'—F3'	5.4 (7)
C5—C6—C7—C8	18.0 (3)	C9—C8—C7'—F3'	−106.0 (7)
C7'—C6—C7—C8	−79.5 (10)	C7—C8—C7'—F3'	168.0 (14)
F3—C7—C8—F5	88.4 (5)	F5—C8—C7'—C6	−131.2 (5)
F4—C7—C8—F5	−31.1 (4)	F6—C8—C7'—C6	120.7 (5)
C6—C7—C8—F5	−147.2 (3)	C9—C8—C7'—C6	9.3 (7)
F3—C7—C8—F6	−26.5 (5)	C7—C8—C7'—C6	−76.6 (10)
F4—C7—C8—F6	−146.0 (3)	F1—C6—C7'—F4'	−0.8 (12)
C6—C7—C8—F6	97.9 (3)	F2—C6—C7'—F4'	111.2 (10)
F3—C7—C8—C9	−144.9 (4)	C5—C6—C7'—F4'	−134.5 (10)
F4—C7—C8—C9	95.6 (3)	C7—C6—C7'—F4'	−46.4 (12)
C6—C7—C8—C9	−20.5 (4)	F1—C6—C7'—F3'	−131.7 (8)
F3—C7—C8—C7'	−44.8 (10)	F2—C6—C7'—F3'	−19.7 (9)
F4—C7—C8—C7'	−164.3 (11)	C5—C6—C7'—F3'	94.7 (9)
C6—C7—C8—C7'	79.6 (10)	C7—C6—C7'—F3'	−177.3 (15)
C3—C5—C9—C10	−11.3 (5)	F1—C6—C7'—C8	122.1 (5)
C6—C5—C9—C10	175.0 (3)	F2—C6—C7'—C8	−125.8 (5)
C3—C5—C9—C8	169.5 (3)	C5—C6—C7'—C8	−11.5 (7)
C6—C5—C9—C8	−4.2 (3)	C7—C6—C7'—C8	76.6 (10)
